# Defective postreplication repair of UV photoproducts in melanoma: a mutator phenotype

**DOI:** 10.1002/1878-0261.12612

**Published:** 2019-12-31

**Authors:** Douglas E. Brash, Michael M. Seidman

**Affiliations:** ^1^ Departments of Therapeutic Radiology and Dermatology and Yale Cancer Center Yale School of Medicine New Haven CT USA; ^2^ Laboratory of Molecular Gerontology National Institute on Aging NIH Baltimore MD USA

## Abstract

In this issue, the Gabrielli laboratory and collaborators address the bulky CPD lesions created in DNA when UV joins two adjacent pyrimidines (thymine or cytosine), leading to skin cancers such as melanoma (Pavey S *et al*. (2019) Mol Oncol). Our understanding of postreplication repair mechanisms for bulky lesions has lagged, and the newly reported predominance of translational control in the UV response has important implications. Image taken from https://www.flickr.com/photos/atul666/2059154608 by brx0, licensed under CC BY‐SA 2.0. https://doi.org/10.1002/1878-0261.12601

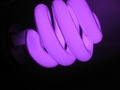

**Abbreviation**
CPD: cyclobutane pyrimidine dimer

It is an old chestnut that mutations arise during DNA replication. But when DNA polymerase encounters an obstacle – DNA secondary structure, a chemical adduct, or an ultraviolet‐light (UV)‐induced cyclobutane pyrimidine dimer (CPD) – it actually has three options: It can arrest in place, perhaps with the other DNA strand spooling out single‐stranded DNA and possibly leading to cell death; it, or a specialized DNA polymerase, can execute translesion synthesis by inserting a base, often an incorrect one; or it can skip over the obstruction, leaving a gap, and continue on (Rupp and Howard‐Flanders, 1968). The translesion synthesis option is well studied (Waters *et al.*, 2009), but the gap option happens more often than what is generally appreciated. These gaps must then be repaired in G2, after DNA replication has finished. One possibility is that the missing information is read from the newly available chromosome copy, using strand invasion steps that resemble the early stages of homologous recombination (Livneh *et al.*, 2016). Recombinational repair is usually studied in connection with DNA double‐strand breaks, such as those made by ionizing radiation (Friedberg *et al.*, 2005). The involvement of genes, such as those belonging to the *BRCA* and *FANC* families, has implicated DNA recombination at strand breaks as a postreplication repair mechanism that, on balance, reduces the frequency of cancers of the breast and ovary (Zhao *et al.*, 2019). In the UV case, the original CPD must then also be repaired. In melanocytes, these processes must work quickly because UV stimulates melanocyte proliferation (van Schanke *et al.*, 2005).

In this issue, the Gabrielli laboratory and collaborators address the bulky CPD lesions created in DNA when UV joins two adjacent pyrimidines (thymine or cytosine), leading to skin cancers such as melanoma (Pavey *et al.*, 2019). CPDs are also considered a model for bulky lesions left by cigarette smoke or chemotherapy drugs such as *cis*‐platinum and psoralen. Postreplication repair is a tricky subject on which to do conclusive experiments, requiring virtuoso manipulation of S phase, G2 phase, and cell signaling. Moreover, manipulating these experimentally can have side effects such as apoptosis. All these points must be tested and the suitable controls included in experiments. As a result, our understanding of postreplication repair mechanisms for bulky lesions has lagged. The paper's authors previously found that, although melanoma cell lines exhibit normal excision repair of CPDs, they are commonly defective in postreplication repair and accumulate single‐strand gaps at CPDs (Pavey et al., 2013; Wigan *et al.*, 2012). The single‐stranded DNA then triggers a G2 checkpoint pathway containing familiar members, such as replication protein A, ATR kinase, and serine/threonine‐protein kinase CHK1, causing G2 arrest until repair proteins arrive, and less familiar members, such as E3 ubiquitin–protein ligase RAD18, which recruits translesion polymerases. But what proteins participate in this checkpoint and repair process, and which underlie the melanoma defect?

In the present study, the researchers examined the polysome profile of melanoma cells to identify genes translated – not transcribed – as part of the cell's UV‐induced checkpoint and repair response. This analysis produced several striking findings. First, translation variation is more important in the UV response than transcriptional variation, with 85% of the G2 transcripts unique to UV‐irradiated cells being regulated only at the translational level. Second, testing the function of these genes using siRNA revealed five clusters of gene functions: response to single‐stranded DNA (e.g., *ATR*, *CHK1*); global genomic nucleotide excision repair (*XPC*, *DDB1*, *DDB2*); recombinational repair (*BRCA1*, *RAD51*, and others); transcription signaling, including cell cycle regulators (*STAT3*, *JUN*, *CDKN1A*, *CCND1*); and the *MASTL* pathway. MASTL, the investigators found, controls progression from G2 arrest into mitosis, but the direction of its effect and its position in the pathway depend on whether it is depleted transiently or constitutively. Third, bioinformatics revealed that both the DNA repair and checkpoint genes contributed to the CPD‐induced UV signature mutations (C → T mutations at pyrimidine–pyrimidine sites) that are extremely frequent in melanomas (Alexandrov *et al.*, 2013; Brash, 2015). The top three checkpoint genes were *MASTL* and two other members of the *MASTL* pathway.

The reported predominance of translational control in the UV response fits with the findings of a recent search for polymorphisms that accelerate murine melanomas induced by a single neonatal UV dose: Ferguson et al reported that a component of the ribosome was the mostly affected gene (Ferguson *et al.*, 2019). Similarly, Premi *et al.* (2019) reported that UV‐sensitive hyperhotspots for CPD formation in melanocytes are typically located in regulatory regions of genes for ribosomes and other elements of RNA metabolism. The involvement of both gap‐filling pathways and nucleotide excision repair is consistent with the timing and behavior of this checkpoint: Completion of gap‐filling converts single‐ to double‐strand DNA, preventing single‐strand nucleases from creating a disastrous double‐strand break at the gap and enabling the nucleotide excision machinery to excise the CPD and several nucleotides on either side. One guesses that translesion polymerases and template switching mechanisms compete for gaps – whatever it takes to finish the job before mitosis begins. This ecosystem leaves the question: Why does a failed G2 checkpoint generate more UV signature mutations than ordinary S‐phase translesion synthesis? Given that MASTL regulates the G2 → M transition, perhaps the daughter cell inherits the gaps but, without a sister chromatid, it can use only translesion synthesis.

This study has broader implications as well. Assuming that most of a patient's relevant sunlight exposure occurred before the melanoma appeared, either the melanoma patient or an abnormal founder cell had a defect in postreplication repair of CPDs – a mutator phenotype (Loeb, 2016). This circumstance could explain the curious fact that gene promoter mutations in melanomas are 100% UV signature mutations (Fredriksson *et al.*, 2017; Hayward *et al.*, 2017), rather than the 70–80% expected from UV exposure to normal cells (Brash, 2015). If that postreplication repair deficiency were a property of the patient, susceptible individuals should be identifiable and could be advised about sunlight protection and be monitored for early, curable cancers. Whether this susceptibility reflects an allele of a particular gene for the checkpoint or postreplication repair, or is a required phenotype reached by mutations in different genes in different patients, remains to be ascertained.

## Conflict of interest

The authors declare no conflict of interest.
